# Spontaneous Coronary Artery Dissection Associated With Infertility Treatment

**DOI:** 10.7759/cureus.29587

**Published:** 2022-09-26

**Authors:** Cecilia Iyasere, Neelam Potdar

**Affiliations:** 1 Health Sciences, University of Leicester, Leicester, GBR; 2 Obstetrics and Gynaecology, University Hospitals of Leicester NHS Trust, Leicester, GBR

**Keywords:** assisted conception, in vitro fertilisation, subfertility, cardiovascular disease, spontaneous coronary artery dissection

## Abstract

Assisted conception involving hormonals is a risk factor for spontaneous coronary artery dissection (SCAD), and pregnant women with spontaneous coronary artery dissection are more likely to have had treatment for subfertility. Increasingly, there is a risk of maternal death in women after assisted conception, and so, the need to assess the cardiovascular sequelae after assisted conception is imperative. This is an illustrative case of spontaneous coronary artery dissection shortly after a repeat cycle of in vitro fertilisation (IVF).

The aetiology of spontaneous coronary artery dissection is believed to be multi-factorial, affecting mostly young women, a population similar to women requiring assisted conception. The oestrogen and progesterone used in in vitro fertilisation are believed to trigger structural weakening in the coronary blood vessels, leading to vascular rupture. Repeat in vitro fertilisation cycles and successful conception are thought to increase spontaneous coronary artery dissection risk by increasing hormonal exposure.

The management of spontaneous coronary artery dissection is dependent on if pregnancy has been achieved or not, and a multi-disciplinary approach to its management is essential.

More research is needed to identify women at higher risk of this life-threatening event.

## Introduction

Cardiovascular disease is a leading cause of maternal death in the UK, and 7.2% of these deaths were due to spontaneous coronary artery dissection (SCAD) [1]. Of all maternal deaths of a cardiovascular cause, 7% were in women who had assisted conception, and so, there is a greater need to review cardiovascular sequelae following assisted conception [1]. This case report focuses on the links between assisted conception (in vitro fertilisation (IVF)) and spontaneous coronary artery dissection (SCAD).

SCAD is a rare non-traumatic spontaneously occurring tear in the coronary artery wall, with an unclear aetiology. Its likely aetiology is multi-factorial, involving hormonal, environmental and genetic factors. SCAD is a rare cause of acute coronary syndrome (ACS) with an incidence of 0.1%-4% [2]. It is often an underdiagnosed cardiac event, seen mostly in healthy women under the age of 50, and is responsible for 24% of ACS in women [3]. It is not usually associated with known risk factors for cardiovascular disease, and therefore, its management varies. It has been associated with pregnancy, hormonal treatments (oral contraceptive pills, progesterone, human chorionic gonadotropin (HCG) and testosterone), extreme emotional stress (such as bereavement), extreme exercise, certain connective tissue disorders (fibromuscular dysplasia (FMD)), sympathomimetic drug use (amphetamines and cocaine) and activities causing sustained Valsalva manoeuvre [3]. The clinical presentation can range from completely asymptomatic to sudden cardiac death.

## Case presentation

This is an illustrative case of a 32-year-old female undergoing in vitro fertilisation (IVF) for primary infertility, with a history of familial hypercholesterolaemia and trivial mitral regurgitation diagnosed on an echocardiogram. She had a failed IVF cycle the previous year, which ended in failure of fertilisation. She underwent a second IVF cycle with agonist protocol (Buserelin, a gonadotropin-releasing hormone analogue and human menopausal gonadotropin) for controlled ovarian stimulation. She had seven oocytes retrieved and underwent a fresh single embryo transfer. She was commenced on progesterone vaginal pessaries 400 mg twice a day for luteal phase support.

**Figure 1:**
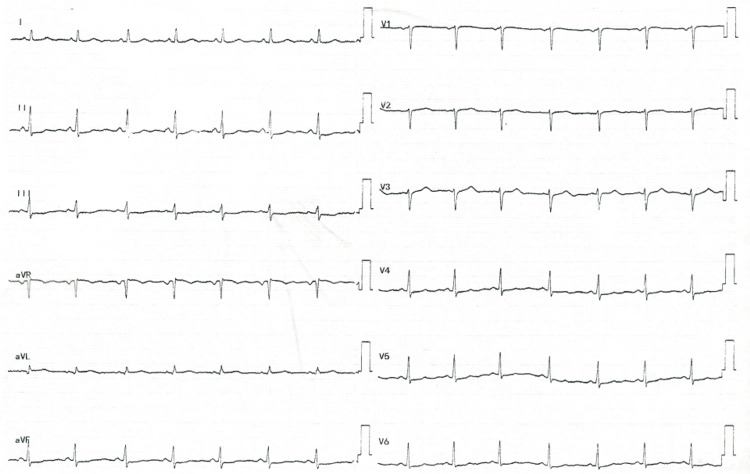
Electrocardiogram showing sinus rhythm

Ten days after embryo transfer, she presented to accident and emergency with sudden-onset back pain and one episode of loss of consciousness. Her last gonadotropin injection was 15 days prior. Her admission electrocardiogram (EKG) showed sinus rhythm (Figure 1). Her troponin levels were 36,960 ng/mL, and she had D-dimers of 2.6 ug/mL fibrinogen equivalent units (FEU) (Table 1).

**Table 1:** Laboratory results FEU: fibrinogen equivalent units

Test	Result	Normal range
D-dimer	2.6 ug/mL FEU	0-0.50 ug/mL FEU
Troponin-I	36,960 ng/mL	0-0.04 ng/mL
Creatinine kinase	1,546 IU/L	25-200 IU/L

**Figure 2:**
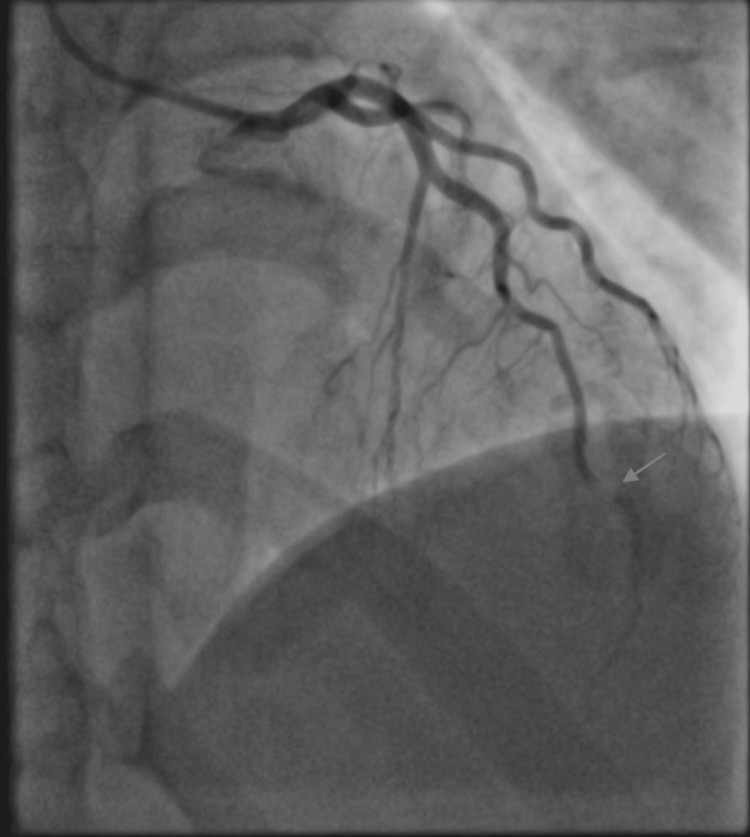
Coronary angiogram showing a dissection in the apical left anterior descending coronary artery

Differential diagnoses at presentation included ovarian hyperstimulation syndrome, pulmonary embolism and aortic dissection. Coronary angiography showed spontaneous coronary dissection of the apical left anterior descending coronary artery (Figure 2).

**Figure 3:**
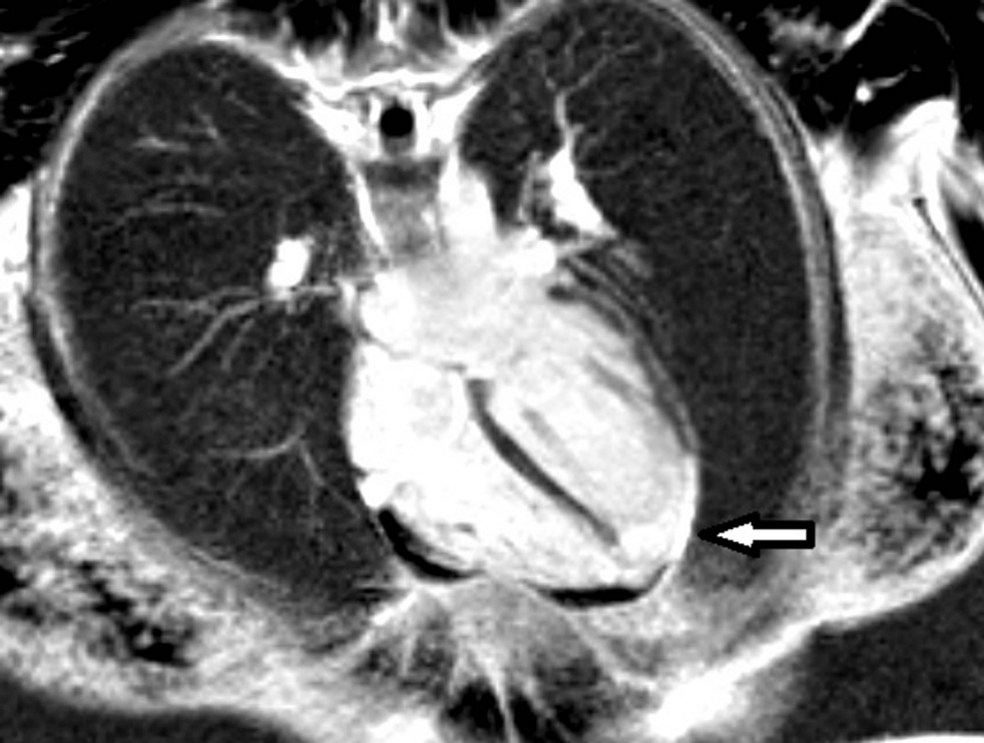
Distal left anterior descending territory infarction with scarring (arrow)

A bubble echo study excluded an interatrial shunt and showed a hypokinetic apex with preserved left ventricular function (left ventricular ejection fraction (LVEF) of 52%). She was placed on dual antiplatelet treatment and discharged on bisoprolol and aspirin. Eight days later, she had a positive pregnancy test and was managed in pregnancy by a multi-disciplinary team of cardiologists, obstetricians and anaesthetists, with clinical reviews and foetal growth scans every four weeks. The bisoprolol and aspirin were continued in pregnancy at doses of 1.25 mg and 150 mg, respectively, with the plan to drop the dose of aspirin to 75 mg post-natally. At 28 weeks, she had magnetic resonance imaging of her heart with late gadolinium enhancement and a magnetic resonance angiogram of her whole aorta to assess the extent of myocardial injury and exclude fibromuscular dysplasia (FMD). FMD was excluded, but a distal left anterior descending territory infarction was seen, with scarring (Figure 3). However, her left ventricular ejection fraction was preserved.

Her intrapartum care plan was for an early epidural in labour and to avoid ergometrine as a uterotonic for delivery of the placenta. She was induced at 37 weeks for severe foetal growth restriction and had a vaginal delivery with the baby weighing 2,100 g at birth.

## Discussion

Spontaneous coronary dissection in association with assisted conception treatment is rare. A thorough literature search has shown sparse information about SCAD occurring shortly after IVF and the impact of hormones used for controlled ovarian stimulation and progesterone pessaries. Literature, however, shows plentiful evidence of SCAD linked with late pregnancy, especially in the post-partum period. Other known risk factors for SCAD include oral contraceptive pills, progesterone, HCG, testosterone, extreme stress and exercise, certain connective tissue disorders (fibromuscular dysplasia), sympathomimetic drug use (such as amphetamines and cocaine) and activities causing sustained Valsalva manoeuvre.

Hormone-mediated connective tissue changes are thought to occur in the coronary arteries, leading to loss of elastic fibre structure and collagen degeneration. Similarly, it is suspected that the hormonal changes occurring during IVF treatment and pregnancy may cause weakening in the coronary blood vessels. With IVF, the follicles that ovulate undergo luteinisation, producing progesterone and oestrogen. Progesterone support given after egg retrieval and produced by the early trophoblast further increases circulating progesterone levels. The hypothesis is that the oestrogen and progesterone hormone receptors found in the coronary blood vessels cause an architectural change in the vascular wall, as it occurs in other blood vessels, leading to weakening and vascular rupture [3]. High levels of progesterone reduce the stretch of elastic fibres [4,5] and decrease the levels of ground substance, whilst oestrogen can reduce the amount of structural support in blood vessels by causing the release of metalloproteinases [4-6]. One could extrapolate that the use of hormones in IVF treatment could predispose to SCAD through a similar mechanism of action. Significant haemodynamic changes that occur during the first weeks of pregnancy would increase shearing forces in the coronary artery, therefore increasing this predisposition. Women undergoing more than one cycle of IVF would therefore be exposed repeatedly to the hormone-induced changes, with the potential outcome of SCAD, as occurred in the illustrated case. This risk would be further amplified in the presence of other risk factors for SCAD, such as if pregnancy is achieved. However, she did not have a positive pregnancy test until after the SCAD. Hence, we believe that the IVF was a major contributor.

A study looking at SCAD in pregnant women in the USA found that compared with other women of childbearing age in the US population, patients with SCAD were more frequently treated for subfertility (28% versus 12%). Of the pregnant women with SCAD, 9% had IVF. Women with SCAD in pregnancy were also more likely to have a history of single or combination subfertility treatment (28% versus 16%). The use of selective oestrogen receptor modulators was reported in 15%, gonadotropin therapy in 9%, aromatase inhibitors in 4%, leuprolide in 2% and progesterone in 6% [7]. This study shows an association between IVF and SCAD, although there is no evidence of direct causality.

New research is looking into the possibility of a genetic element to SCAD [8], and cases of familial SCAD have been reported [9].

Symptoms

SCAD presentation is often acute with symptoms including chest, back, upper mandibular and upper arm pain. Other symptoms include nausea, vomiting, dyspnoea, a popping sensation in the chest and numbness in the extremities. Clinical findings included ST-segment elevation (57% versus 43%) compared with non-ST-segment elevation, ventricular arrhythmias and cardiac arrest. SCAD was found to occur most commonly in the left anterior descending artery [7,10] and in multiple vessels.

Diagnosis

Following tests such as electrocardiogram and troponin levels, coronary angiography should be the first-line diagnostic tool, especially because of its easy accessibility. Other imaging methods for diagnosis, especially with inconclusive angiography, include intravascular ultrasound and optical coherence tomography, which can be used to increase visualisation and therefore aid in the diagnosis of SCAD.

Treatment

The initial management is similar to that of acute coronary syndrome (ACS), and subsequently, it is dependent on haemodynamic stability and whether pregnancy has been achieved. A multi-disciplinary approach is required between the cardiologists, reproductive medicine specialists and obstetricians where treatment has achieved pregnancy.

Conservative Treatment

Repeat angiography following treatment has shown 70% spontaneous healing in 70%-97% of SCAD lesions [11,12]. However, pregnant patients with SCAD are less likely to spontaneously heal and are more likely to suffer SCAD progression than non-pregnant patients. Therefore, conservative management may be an option in haemodynamically stable patients with close in-patient monitoring.

Medical Treatment

The mainstay for medical treatment includes aspirin, low-molecular-weight heparin, beta blockers+/-clopidogrel, angiotensin-converting enzyme (ACE) inhibitors, angiotensin receptor blockers and statins. Medication such as clopidogrel, ACE inhibitors and angiotensin receptor blockers need to be avoided in pregnant women due to a risk of teratogenicity and insufficient evidence on foetal safety. Aspirin is safe in pregnancy in doses up to 150 mg/day, and beta blocker use is shown to reduce the reoccurrence of SCAD [13]. Caution is advised with the use of beta blockers in pregnancy due to the risk of foetal growth restriction, although bisoprolol appears to have the least risk [14,15].

Percutaneous coronary intervention (PCI) is a treatment option; however, in pregnancy, it carries the risk of foetal exposure to radiation; the mean radiation exposure is 3 mGy, and radiation scatter from the irradiated area cannot be prevented even with abdominopelvic shielding. While pregnancy is not a contraindication to PCI, there is a potential risk of teratogenicity especially in early pregnancy [16-18]. PCI can also lead to the extension of the dissection and propagation of SCAD [13].

Coronary bypass grafting has shown no increased risk of maternal mortality compared to the non-pregnant population but does carry a 20% risk of pregnancy loss [19].

## Conclusions

Little is presently known about SCAD in women undergoing IVF treatment, and further research is required to identify women at risk of this life-threatening cardiac event, as well as to improve the outcomes in the at-risk cohort. A family history of early, unexplained deaths or two or more closely related female members of a family with any history of SCAD or myocardial infarction should be taken in women who are offered assisted conception and should prompt the possibility of referral to a geneticist. Counselling amongst this group of women may help reduce the risk of death in these women by creating an awareness of the possible cardiovascular risks they face with IVF. Cardiovascular assessment prior to assisted conception may help in identifying women who are at higher risk of atherosclerotic and non-atherosclerotic ACS, and research is needed to identify possible risk factors for SCAD. Repeated exposure to assisted reproductive technology in some women may also increase the risk of SCAD. Multi-disciplinary care is required for women who develop SCAD during pregnancy with regular maternal and foetal monitoring.
